# Short-Term Exposure to High-Temperature Water Causes a Shift in the Microbiome of the Common Aquarium Sponge *Lendenfeldia chondrodes*

**DOI:** 10.1007/s00248-020-01556-z

**Published:** 2020-08-07

**Authors:** Sergio Vargas, Laura Leiva, Gert Wörheide

**Affiliations:** 1 Department of Earth and Environmental Sciences, Paleontology & Geobiology, Ludwig-Maximilians-Universität München, Richard-Wagner-Str. 10, 80333 Munich, Germany; 2 Present Address: Biologische Anstalt Helgoland, Shelf Sea System Ecology, Alfred-Wegener-Institut Helmholtz-Zentrum für Polar- und Meeresforschung, 27498 Helgoland, Germany; 3 GeoBio-Center, Ludwig-Maximilians-Universität München, Richard-Wagner-Str. 10, 80333 Munich, Germany; 4 SNSB – Bayerische Staatssammlung für Paläontologie und Geologie, Richard-Wagner-Str. 10, 80333 Munich, Germany

**Keywords:** Climate change, Cyanobacteria, *Lendenfeldia chondrodes*, Microbiome, Sponges, Porifera

## Abstract

Marine sponges harbor diverse microbiomes that contribute to their energetic and metabolic needs. Although numerous studies on sponge microbial diversity exist, relatively few focused on sponge microbial community changes under different sources of environmental stress. In this study, we assess the impact of elevated seawater temperature on the microbiome of cultured *Lendenfeldia chondrodes*, a coral reef sponge commonly found in marine aquaria. *Lendenfeldia chondrodes* exhibits high thermal tolerance showing no evidence of tissue damage or bleaching at 5 °C above control water temperature (26 °C). High-throughput sequencing of the bacterial 16S rRNA V4 region revealed a response of the microbiome of *L. chondrodes* to short-term exposure to elevated seawater temperature. Shifts in abundance and richness of the dominant bacterial phyla found in the microbiome of this species, namely Proteobacteria, Cyanobacteria, Planctomycetes, and Bacteroidetes, characterized this response. The observed resilience of *L. chondrodes* and the responsiveness of its microbiome to short-term increases in seawater temperature suggest that this holobiont may be capable of acclimating to anthropogenic-driven sublethal environmental stress via a re-accommodation of its associated bacterial community. This sheds a new light on the potential for resilience of some sponges to increasing surface seawater temperatures and associated projected regime shifts in coral reefs.

**Electronic supplementary material:**

The online version of this article (10.1007/s00248-020-01556-z) contains supplementary material, which is available to authorized users.

## Introduction

By the end of this century, moderate climate change scenarios predict the surface temperature of the globe to be over 1.5 °C higher than in 1850–1900, and the oceans are expected to keep warming throughout the twenty-first century [1]. Extreme events, such as the marine heatwave recorded on the Great Barrier Reef in 2016, are expected to become more common under those scenarios [1], and their detrimental impact on coastal ecosystems will undoubtedly continue to cause significant shifts in the species composition of marine communities [e.g., 2].

An increase in seawater temperature of one to two degrees over maximum summer temperatures approaches the upper tolerance level of important coastal ecosystem engineers, like corals [3] and some sponges tested to date [4–9]. The reef sponge *Rhopaloeides odorabile*, for instance, showed signs of necrosis after 24 h of exposure to seawater temperatures of 33 °C, only about two to four degrees above the mean highest summer temperature at the site of collection [4]. At this temperature, the bacterial community associated with *R. odorabile* suffers irreversible compositional shifts likely caused by a “breakdown” of the sponge symbiotic functions and the colonization of the sponge by opportunistic, potentially pathogenic bacteria [4]. Simister et al. [10] reported similar results. These authors found a shift in the bacterial community associated with *R. odorabile* after exposing this sponge to a seawater temperature of 32 °C. In both experiments, the authors reported a highly stable microbiome at temperatures below that upper limit [4, 10].

Beside *R. odorabile*, several reports of a highly stable sponge microbiome at temperatures below a specific threshold exists. The microbiome of the Australian reef sponge *Ianthella basta* is stable across a latitudinal gradient of over 1000 km and at temperatures up to 31 °C, with microbiome changes only evident after exposure of the sponges to lethal water temperatures (> 32 °C) [5]. Also, the microbiome of the Mediterranean species *Ircinia fasciculata* and *I. oros* appears to be resistant to sublethal heat stress [11]. However, once the water temperature exceeds an upper limit, the microbiome of *I. fasciculata* changes, usually preceding the onset of disease and, eventually, the death of the affected sponges [9]. Perhaps unexpectedly, the microbiome of the boreal sponge *Geodia barreti* was also not affected by exposure to acute thermal stress (i.e., 5 °C above “normal” environmental seawater temperature) for up to 14 days [12]. Thus, the resilience of the sponge microbiome to sublethal thermal stress does not appear to be determined by the geographic area where the sponges occur.

Throughout its ~ 600 million years long evolutionary history [13, 14]‚ Phylum Porifera has evolved to become a significant part of benthic communities worldwide [15], occurring from the tropics to the polar regions in habitats ranging from the eulittoral to the hadal zone [15–17] and playing essential roles in nutrient cycling in these ecosystems [15, 18–20]. The diverse functional roles and ecological flexibility of sponges partly result from their diverse microbiomes [21–23], and although many sponge species have highly stable microbiomes regardless of the environment [24, 25], environmental changes can trigger compositional shifts in sponge microbiomes [26] that can affect the fitness of these holobionts. Thus, investigating the effect of environmental change on the sponge microbiome is pivotal to our understanding of the consequences of climate change upon sponge holobionts and the benthic communities where these organisms occur.

*Lendenfeldia chondrodes*, a common (blue) aquarium sponge, is an easy-to-culture, promising (cyano)sponge holobiont model [27]. Here, we characterized the microbiome of *L. chondrodes* using high-throughput 16S rRNA sequencing and examined the effect of a rapid, short-term increase in sea surface temperature (SST) on the diversity and community composition of the microbiome of this species.

## Material and Methods

### General Description of the Aquarium System and the Heat Shock Experiments

We cultured purple and green morphs of *L. chondrodes* (Suppl. Fig. 1) in a 360-L marine aquarium at the Molecular Geobiology & Paleobiology Laboratory of the Dept. of Earth and Environmental Sciences of the Ludwig-Maximilians-Universität München, under a 12 h day, 12 h night cycle controlled by GHL Mitras LX 6200-HV LED lights. Light intensity at the water surface is ~ 10 klux. Based on hourly measurements over one year (2017), average water temperature and pH are 24.92 ± 0.24 °C and 8.30 ± 0.14, respectively. Based on weakly measurements over one year (2017), the average PO_4_^3−^, NO_2_^−^, and NO_3_^−^ concentrations in the water are 0.092 ± 0.071 mg/L, 0.014 ± 0.072 mg/L, and 2.681 ± 3.882 mg/L; the concentration of NH_3_/NH_4_^+^ in the water was consistently below detection (i.e., < 0.05 mg/L). These values are stable in time as shown by continued water sampling.

For the heat shock experiments, we cut circular sections of the sponges (12 per color morph) with a medical biopsy puncher (0.5 cm diameter) and let them heal in the main aquarium (see above) for one week. We then randomly distributed the sponge explants in six 10-L experimental tanks (i.e., two explants per color morph per tank) filled with ~ 6 L artificial seawater and partially immersed in the 360-L aquarium described above. Water evaporation in the 10-L tanks was compensated every day with water filtered by reverse osmosis. To provide an adequate water mixing, we used a submersible water pump in each tank (300 L/h; Eheim, Germany). After an acclimation period of four days in the experimental tanks, we randomly selected three tanks and used a TetraTec HT 25 heating device (Eheim, Germany) to gradually increased the water temperature over a period of five days (~ 1 °C per day) from ~ 25.4 to ~ 31 °C. During the course of the experiment, we monitored the water temperature every minute using PCE-PHD 1 dataloggers (PCE Instruments, Germany) and, additionally, we manually measured the temperature of all aquaria twice a day (in the morning and the evening) using a regular thermometer (TFA, accuracy ± 0.5 °C). Throughout the experiment, water conductivity, pH, density, redox potential, and nutrient profile in the 10-L tanks were similar to the values observed for the main tank. The water temperature of the control tanks was ~ 25.4 °C over the whole experimental period. At the end of the experiment, we rinsed all explants with sterile-filtered artificial seawater, fixed them in 99% ethanol, and kept them at − 20 °C until further processing.

### High-Throughput Sequencing Bacterial 16S rRNA V4 Region of Control and Treated *L. chondrodes*

We extracted genomic DNA using the Macherey-Nagel NucleoSpin DNA extraction kit, following the protocol provided by the manufacturer, and amplified the V4 region of the 16S rRNA gene using barcoded forward (515fB) and reverse (806rB) primers as described in [28]. In addition to the aquarium samples, and for comparison purposes only, we also sequenced a single specimen casually collected off at St John’s Island, Singapore, at 3-m depth, and determined to be *L. chondrodes*. Before sequencing, we visualized PCR products on 1% agarose gels and extracted bands of the expected size (ca. 380 bp) with the Qiagen QIAquick Gel Extraction Kit. We then quantified all gel-extracted PCR products on a Qubit 3.0 fluorometer (Life Technologies, Grand Island, NY) and diluted them, if necessary, to achieve a final concentration of 1 nM before equimolar pooling. We sequenced the 16S rRNA amplicon pool on an Illumina MiniSeq in mid-output 300PE mode [see 28 for details]. We also determined the bacterial load of each extraction using real-time quantitative PCR (RT-qPCR). For this, using the same V4 region of the 16S rRNA forward (i.e., 515fB) and reverse (i.e., 806rB) primers but without the barcode+Illumina adapter extension, we amplified an untreated *L. chondrodes* sample, gel-extracted the resulting amplicon, and quantified it on a Qubit 3.0 fluorometer (Life Technologies, Grand Island, NY). We then estimated the number of V4 16S rRNA molecules per μL purified amplicon using the formula N_molecules = (ng_DNA*6.022 × 10^23^)/(amplicon_length*1 × 10^9^*650) with the expected amplicon length (i.e., 380 bp). For the calculation of the bacterial load per sample, we used the quantified amplicon to generate a standard dilution series ranging from 10^2^ to 10^8^ V4 16S rRNA molecules/μL and used this series as the standard curve of a qPCR absolute quantification. For quantification, we used a three-step PCR with an initial denaturation step of 95 °C for 3 min followed by up to 40 cycles consisting of 30 s denaturation at 95 °C, 30 s annealing at 50 °C, and 30 s extension at 72 °C. We performed a melting analysis after all qPCRs and used technical duplicates for both samples and standard dilutions. We also included duplicated no template samples as negative controls. After qPCR, we estimated the primer efficiency for each sample and repeated samples with efficiencies lower than 1.8. We used the bacterial load of each sample to provide bacterial load “corrected,” absolute OTU counts after amplicon sequencing (see below) and used the corrected datasets to corroborate the results obtained using the uncorrected dataset. Unless otherwise specified in the text, we used the uncorrected counts for all analyses presented in this study. The reads generated are publicly available at the European Nucleotide Archive under study accession number PRJEB35927.

### Bioinformatic Analysis of the 16S rRNA Amplicon Data

After demultiplexing, we processed the resulting sequences using vsearch [29]. Briefly, we assembled each sequence pair allowing a minimum overlap of 45 bp, dereplicated within and across samples, removed singletons and chimeras, and clustered sequences at 97% similarity to derive a “raw” operational taxonomic unit (OTU) counts table. We then used the RT-qPCR data on bacterial load to provide absolute counts per sample for each OTU in the OTU table. For this, briefly, we rescaled the obtained OTU counts to the total number of bacteria per μL DNA. We determined the taxonomic affiliation of all OTUs found in *L. chondrodes* using the SILVA database “Alignment, Classification and Tree Service” (accessed May 22, 2019) and used the OTU count tables and the taxonomy annotations to determine phylum- and OTU-level richness and abundance patterns, and to fit rank-abundance dominance curves to the bacterial community associated with *L. chondrodes*. We also determined the core bacterial community of *L. chondrodes* as those bacterial OTUs present in all sequenced replicates independently of the treatment applied to them.

To assess the beta-diversity of treated vs. control sponges, we used non-metric multidimensional scaling (NMDS) with the Bray-Curtis distance. We also tested for significant effects of heat stress on bacterial composition using canonical correspondence analysis (CCA) with treatment (i.e., control vs. heat stress), color morph (i.e., green vs. purple) and their interaction as response variables. In order to test for a differential response to heat stress of core vs. non-core OTUs, we conducted separate CCAs for these bacterial groups. To better understand the dynamics of the bacterial community in response to heat stress, we identified OTUs gained or lost as a result of the treatment. For core OTUs, we also calculated log-fold changes in heated vs. control samples. Finally, we compared the representative 16S rRNA sequences of the core OTUs against the top 24 most abundant OTUs derived from the single “wild” *L. chondrodes* sample available to us.

We used R with the packages vegan [30] and DESeq2 [31] for the analyses and deposited all scripts and raw data tables used in the project repository (https://gitlab.lrz.de/cbas/CBAS_16S).

## Results

### The Microbiome of *L. chondrodes* Is Rich but Dominated by a Small Number of Bacterial Associates

**Fig. 1:**
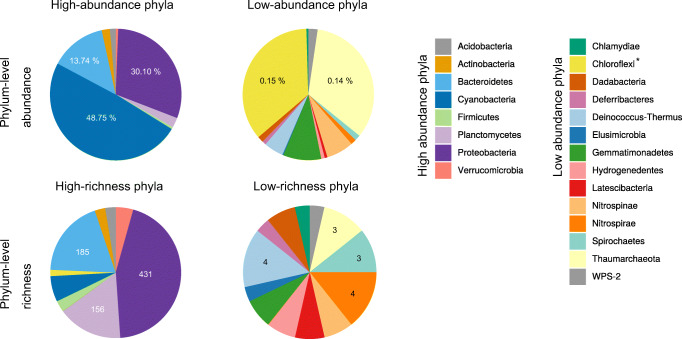
Abundance and richness of bacterial phyla associated with the common aquarium sponge *Lendenfeldia chondrodes*. High-abundance phyla are defined as having at least 1000 16S V4 rRNA read counts. High-richness phyla are those with at least 10 OTUs. Supplementary Figs. 2 and 3 provide phylum-level abundance and richness plots per sample, respectively. Rarefaction curves for each sample are provided in Suppl. Fig. 4

In total, we successfully extracted, amplified, and sequenced a total of 20 samples. These included 6 purple and 6 green control sponges and 5 purple and 3 green treated sponges. Using these samples, we found 1343 OTUs affiliated to 22 bacterial phyla associated with *L. chondrodes*. Phylum Proteobacteria had the highest OTU richness (431 OTUs), followed by Bacteroidetes (185 OTUs) and Planctomycetes (156 OTUs). The remaining 19 phyla had considerably less OTUs assigned to them. For instance, we found 61 and 42 OTUs belonging to the phyla Cyanobacteria and Firmicutes, respectively, and all remaining phyla had 25 or fewer OTUs (Fig. 1). Dominance patterns differed radically from those of richness, with Cyanobacteria dominating the community and accumulating 47% of all the sequenced reads. The phyla Proteobacteria and Bacteroidetes followed in abundance with 29% and 13% of the reads, respectively. The remaining 19 phyla accounted for 11% of all the sequenced reads (Fig. 1). The core bacterial community of *L. chondrodes* is composed of 17 bacterial OTUs belonging to the phyla Acidobacteria, Actinobacteria, Bacteroidetes, Cyanobacteria, Planctomycetes, and Proteobacteria, and showing significant similarity to bacterial sequences derived from other sponges (Suppl. Table 1). These OTUs accounted for 78% of the sequenced reads across all samples. The analysis of a single *L. chondrodes* sample allowed us to compare the microbiome of cultured and wild *L. chondrodes* samples. In wild *L. chondrodes*, we found 62 bacterial OTUs of which the top 24 most abundant OTUs account for 99% of all the sequenced reads. Among these 24 OTUs, we found eight of the core OTUs detected in cultured *L. chondrodes* samples (Table 1). These OTUs had a 100% identity between wild and cultured *L. chondrodes*. In addition, we found that the top two most abundant OTUs, namely one cyanobacterium and one bacteroidetes, in cultured and wild samples of *L. chondrodes* were identical.

**Table 1:** Core bacterial OTUs in wild vs. cultured samples of *L. chondrodes*. The OTU names are arbitrary and given by the OTUs clustering software independently for wild and cultured samples. OTU number roughly reflects the abundance rank, which is provided for wild and cultured samples. OTUs matching or with similar abundance ranks in bold. The taxonomic assignment was conducted independently for each sample and compared. Only OTUs with identical taxonomic assignment and 100% identical V4 16S rRNA sequences are reported

OTUs		Abundance rank		
Wild	Cultured	Wild	Cultured	Taxonomy
OTU_1	OTU_1	1	1	Bacteria;Cyanobacteria;Oxyphotobacteria;Synechococcales;Cyanobiaceae;[Synechococcus] spongiarum group
OTU_2	OTU_2	2	2	Bacteria;Bacteroidetes;Rhodothermia;Rhodothermales;Rhodothermaceae;uncultured
OTU_6	OTU_4	7	4	Bacteria;Proteobacteria;Gammaproteobacteria;Oceanospirillales;Pseudohongiellaceae;Pseudohongiella
OTU_4	OTU_6	5	6	Bacteria;Actinobacteria;Acidimicrobiia;Microtrichales;Microtrichaceae;Sva0996 marine group
OTU_15	OTU_11	18	8	Bacteria;Acidobacteria;Subgroup 9
OTU_3	OTU_18	3	10	Bacteria;Proteobacteria;Gammaproteobacteria;Steroidobacterales;Woeseiaceae;JTB255 marine benthic group
OTU_9	OTU_21	10	11	Bacteria;Proteobacteria;Gammaproteobacteria;UBA10353 marine group
OTU_19	OTU_59	22	15	Bacteria;Proteobacteria;Alphaproteobacteria;Rhodovibrionales;Kiloniellaceae;uncultured

**Fig. 2:**
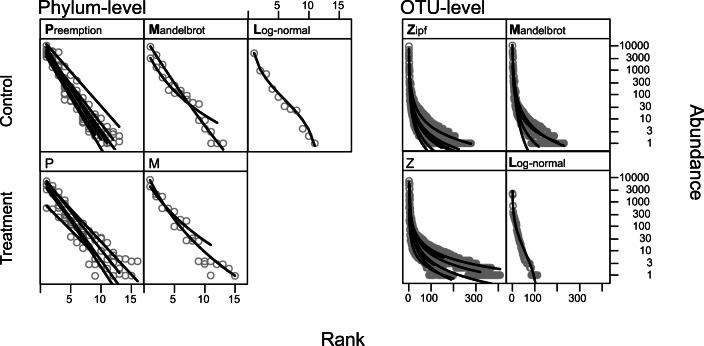
Abundance-rank dominance models fitted to phylum- and OTU-level 16S V4 rRNA counts for control and thermal stress treatments. Lines represent sample replicates; dots are phyla/OTUs. The name of the models are above the curves, and the letter in bold is used as the model abbreviation

As judged by its Akaike information criterion (AIC), the phylum-level best fit abundance-rank dominance model was a geometric series (niche preemption model; [32]) for 15 out of 20 replicates, followed by the Mandelbrot and the lognormal model in four and one replicates, respectively (Fig. 2). Fitting the Mandelbrot model to the phylum-level abundance data was challenging, and some parameters of this model could not be fit. Whenever the full model could be specified, the AIC for the Mandelbrot model was lower than that for the niche preemption model. Generally, the analysis of model deviance across replicates resulted in a non-significant difference between these models (Suppl. Table 2). At OTU-level, the best fit (i.e., lowest AIC) abundance-rank dominance models were the Zipf (15 replicates) or Mandelbrot (four replicates), with only one replicate fitting the lognormal model (Fig. 2). As in the analysis of the phylum-level abundances, an analysis of model deviance revealed that the Zipf and Mandelbrot models were not significantly different (Suppl. Table 3).

### Short-Term Exposure of *L. chondrodes* to Heat Stress Changes Core vs. Non-core Bacteria β-Diversity in a Specific Manner

**Fig. 3:**
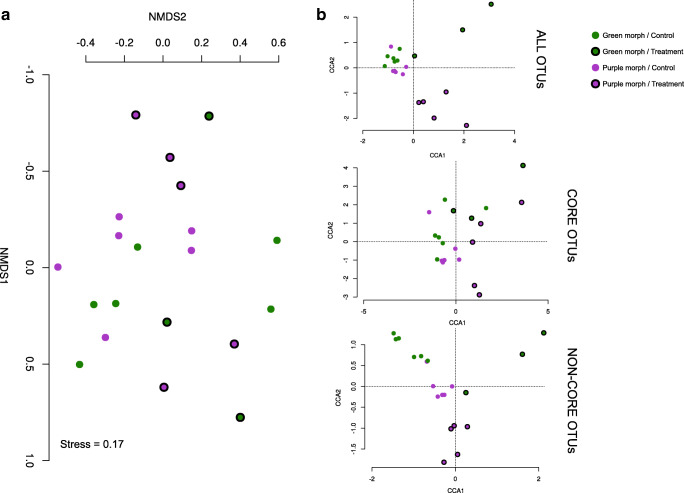
Non-metric multidimensional scaling (**a**) and canonical correspondence (**b**) analysis of the OTU-level composition of the microbiome of *Lendenfeldia chondrodes* in control vs. thermal stress treatments. GC green morph control samples, PC purple morph control samples, GT green morph treatment samples, PT purple morph treatment samples

Heat stress caused a compositional change of the bacterial communities associated with *L. chondrodes*. This effect was somewhat mild in the NMDS plot (Fig. 3a) but marked in the canonical correspondence analysis, where control and treatment samples formed two distinct groups (Fig. 3b). Indeed, we found that the variable “Treatment” (i.e., heat stress vs. control temperature) had a significant effect on bacterial composition while the variable “Color morph” did not; the interaction between these two variables was not significant (Table 2). These results were independent of any filtering (e.g., including only OTUs with more than 50 reads) of the raw count OTU table we used for the analysis. However, these results differed from those we obtained using the bacterial load corrected OTU table. If we used these data, the NMDS analysis did not converge to a solution unless we filtered the OTU table to include only OTUs with more than 50 (absolute) counts. Using this (filtered) table, we also observed a significant effect of the variable “Treatment” on bacterial composition (Suppl. Table 4). In contrast to the analysis of the raw count table, we observed a significant effect of the variable “Color morph” on bacterial composition, while the interaction between these variables was still not significant (Suppl. Table 4).

**Table 2:** Permutation test (999 permutations) for a canonical correspondence analysis of the effect of temperature treatment, sponge color morph, and their interaction on the composition of the microbiome of *Lendenfeldia chondrodes*. Terms added sequentially, first to last

	*df*	Chi-square	*F*	Pr(>*F*)
Treatment	1	0.20026	1.7458	0.008
Color morph	1	0.11300	0.9850	0.492
Treatment × color morph	1	0.13395	1.1677	0.191
Residual	16	1.83543		

**Fig. 4:**
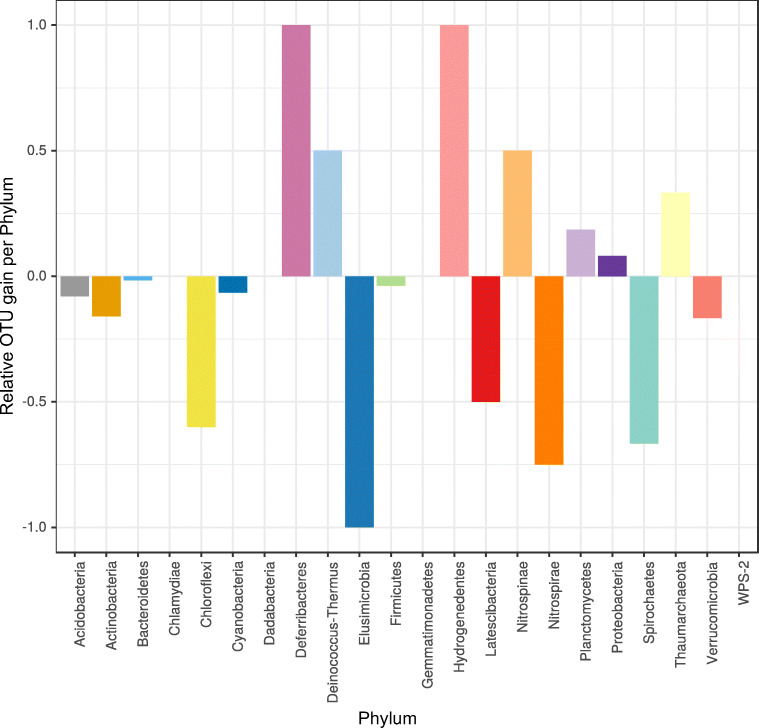
Relative OTU gain/loss per phylum after exposure to thermal stress. Bacterial gain/loss is defined as the appearance/disappearance of an OTU in the treated samples using as reference the composition of control samples

The analysis of core and non-core bacterial OTUs revealed a similar trend and allowed us to better dissect the effect of heat stress on both types (i.e., core and non-core) of bacterial associates. Here again, we detected a significant effect of the heat stress treatment on both core and non-core bacterial associates. However, we only detected a significant effect of sponge color morph for non-core bacterial OTUs. Moreover, this group of OTUs was also affected by the interaction between thermal treatment and sponge color morph. In line with these results, we found that the heat stress treatment induced a change in the community characterized by the net loss of OTUs in treated vs. control sponges (Fig. 4).

Here, 11 out of 22 phyla detected in *L. chondrodes* loss OTUs, while only seven phyla gained new OTUs in response to heat stress. As expected, OTU replacement had a more notable impact on the richness of rare phyla, causing the disappearance of one bacterial phylum (Elusimicrobia) and lowering the richness of other bacterial phyla like Chloroflexi, Latescibacteria, Nitrospirae and, Spirochaetes in treated samples to less than half of the values detected in control sponges. Bacterial phyla benefiting from the heat stress treatment include Deferribacteres and Hydrogenedentes, which were only detectable in heat stress samples, and Deinococcus-Thermus and Nitrospinae, which duplicated their richness in treated vs. control sponges. Thaumarcheota also increased its richness in response to heat stress.

### Heat Stress Affects Different Members of the Core Bacterial Community Associated with *L. chondrodes* in a Specific Manner

**Fig. 5:**
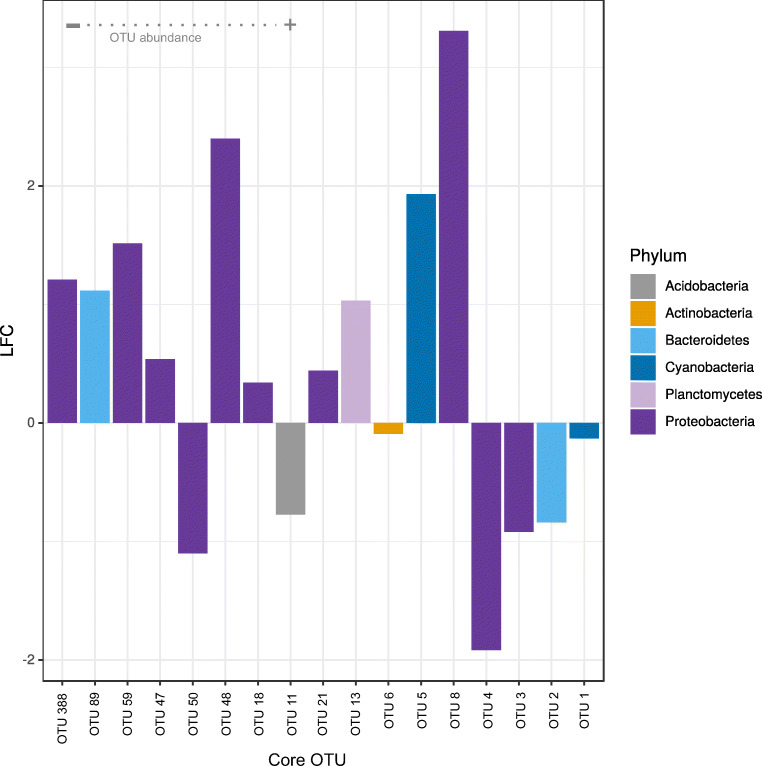
Abundance log2-fold change for core OTUs in *Lendenfeldia chondrodes* in thermal stress vs. control samples. The OTUs are ordered by the relative abundance with OTU 1 and OTU 388 being the OTUs with the highest and lowest abundance, respectively

The analysis of abundance log-fold changes of *L. chondrodes*’ 17 core bacterial OTUs revealed that heat stress had a specific effect on different members of this community. This differential response was not taxon-specific as the abundance of OTUs belonging to the same phylum responded in opposite ways upon exposure to heat stress (Fig. 5). For instance, within Proteobacteria, which with ten OTUs is the richest phylum in the core community of *L. chondrodes*, three OTUs halved (i.e., log-fold change less than − 1) and four OTUs doubled (i.e., log-fold change > 1) their abundances. This trend was also evident in the other phyla with more than one OTU, namely Bacteroidetes and Cyanobacteria. In both phyla, one OTU increased its abundance while the abundance of the remaining OTU assigned to the phylum decreased. The observed change in OTU abundance seems to correlate with the mean overall abundance of these OTUs (Fig. 5). This pattern appears to be general for the core bacterial community. Indeed, the four most and less abundant OTUs decreased and increased their abundance in response to heat stress. Despite this, we did not find a significant correlation between abundance log-fold change and mean overall OTU abundance. Exploration of the regression diagnostics revealed a single, highly influential OTU (i.e., OTU 8, Proteobacteria; Suppl. Fig. 5) with a large residual (e_OTU16 = 3.24 vs. mean_e = − 0.20) and Cook’s D (d_OTU16 = 0.42 vs. mean_d = 0.03). After removing this single OTU from the analysis, abundance log-fold change correlated negatively (Pearson’s *r* = −0.54, Spearman’s rho = − 0.52) with mean overall OTU abundance (Suppl. Fig. 5). This correlation, which was significant independently of the correlation test used, indicates that heat stress induces rare core OTUs to increase their abundance while the abundance of the dominant core OTUs decreases.

## Discussion

Here, we provide the first characterization of the microbiome of *L. chondrodes*, a keratose sponge commonly found in salt-water aquaria around the globe [27]. With over 1300 OTUs belonging in 22 phyla, the microbial community associated with *L. chondrodes* lies well within the ranges of OTU- and phylum-level richness (i.e., 50 to 3820 OTUs, and 13 to 34 phyla, respectively) reported in a microbiome survey of 81 sponge species [33]. The high number of OTUs assigned to phylum Proteobacteria found in *L. chondrodes* also matches previous reports indicating that this phylum is the richest in sponge microbiomes [23]. In *L. chondrodes*, however, the phyla Bacteroidetes and Planctomycetes were also OTU-rich, which contrasts with findings in other sponges [23, 33]. These results highlight the disparity in OTU composition displayed by sponge-associated bacterial communities and further support the proposed importance of host identity in structuring their composition [33]. Reinforcing this idea, we observed similarities between the core microbiome of cultured *L. chondrodes* and the most abundant bacterial OTUs obtained from a single sample of *L. chondrodes* collected in the field. Although the somewhat paradoxical scarcity of field samples of *L. chondrodes*—a species frequently found in aquaria—available for analysis precludes a conclusive assessment of the stability of the microbiome under culture conditions in this species, our data suggest that the microbiome of *L. chondrodes* is stable under culture. We propose that this stability is driven by the dominance of a single cyanobacterium and the abundance distribution among the remaining OTUs.

Sponge microbiomes harbor a mixture of host-generalists and host-specialists and are characterized by reduced core communities of 7 to 20 OTUs [33]. The best-fitting abundance-rank dominance model for the microbiome of *L. chondrodes*, namely the Zipf-Mandelbrot model, allows for a similar interpretation as it points to a microbiome dominated by a small set of host-generalists, likely with few niche requirements, complemented with a low abundance but species-rich assemblage of host-specialists and transient associates with more narrow niches [34, 35]. The original interpretation of the Zipf-Mandelbrot model associates the abundance of each item with a cost, without defining precisely what the cost is [34]. In ecology, a link between the cost of a species and its trophic level exists and implies that autotrophs cost less than heterotrophs [34]. This species-cost scheme predicts a higher abundance of autotrophs in a community, in agreement with our findings in *L. chondrodes*, where a single cyanobacterial OTU dominates the microbiome accounting for almost half of the 16S rRNA reads sequenced. Although at present we cannot fully determine how abundance is apportioned between host- generalist and specialist bacteria in *L. chondrodes*, the fact that the most abundant core bacterium found in this sponge (i.e., *Synechococcus spongiarum*), is widespread among sponges and several other *L. chondrodes*’ core OTUs are similar to bacteria present in other sponges point to a core microbiome largely dominated by host-generalists, as observed in other sponges [33]. It seems of interest to further investigate whether the microbiomes of other cyanosponges behave similarly and fit the Zipf-Mandelbrot model, as this model allows for mechanistic interpretations of the abundance-rank data that could provide insights into the trophic functioning of these bacterial communities.

In contrast with previous reports on several sponge species, we observed a significant compositional change in the microbiome of *L. chondrodes* upon short-term exposure to sublethal heat stress (i.e., from 27 to 31 °C in 1 week). The Caribbean sponge *Xestospongia muta* responded similarly to 12 days of exposure to sublethal heat stress [8]. In both *L. chondrodes* and *X. muta*, the observed microbiome changes did not correlate with any signs of necrosis or discoloration of the sponge tissues. The lack of evident signs of stress at sublethal water temperatures is consistent with previous experiments in other sponge species [4, 5, 10]. However, in those species, sublethal heat stress did not trigger a significant change in the composition of the microbiome. This suggests that despite its convergent nature [33], sponge microbiomes have different levels of resilience to increasing surface seawater temperatures and can show contrasting responses to the same environmental stimulus. Thus, anthropogenic-driven climate change and the associated projected regime shifts in coral reefs [see 36, 37] will result in loser and winner sponge species and will impact the composition of sponge communities in an unpredictable manner.

The response of the *L. chondrodes* microbiome to heat stress involved a re-accommodation of bacterial richness and abundance. In the case of richness, OTU-rich phyla, like Proteobacteria and Planctomycetes, became even richer after the treatment, gaining the largest number of OTUs in absolute terms during the experiment. This trend was, however, not general as other OTU-rich phyla like Bacteroidetes and Cyanobacteria showed the opposite trend, losing OTUs in response to heat stress. In relative terms, however, temperature stress had a more pronounced effect among rare phyla, with some groups doubling or halving its richness and other entirely disappearing from the community. This marked difference in the response of core and non-core OTUs to the increase in water temperature was evident in the canonical correspondence analyses done independently on these two OTU groups (see Fig. 3b). It is likely that the CCA results obtained from the analysis of the entire community are driven by the larger effect of the treatment on non-core OTUs, as rare taxa can have a high influence on the correspondence analysis results [38]. The less clear separation of control and treatment communities in the (unconstrained) NMDS analysis (Fig. 3a), which should be less affected by changes among rare OTUs, likely reflects the compositional stability of the core bacterial community of *L. chondrodes* to the treatment. Given the lack of knowledge on this symbiotic system, and generally on the specific role of the rare microbial biosphere in the functioning of the community, linking the effects of the sudden bacterial OTU gain or loss caused by heat stress with any functional changes of the *L. chondrodes* microbiome remains challenging. The development of methods to assess changes in gene expression at the holobiont level appears thus imperative to further understand *L. chondrodes*’ interactions with its microbiome.

In sponge microbiomes, density-dependent processes determine the abundance of particular OTUs in the core community [33]. Similarly, short-term exposure to sublethal heat stress affected the abundances of core OTUs in a density-dependent manner, causing an increase and a reduction of the population size of the top four less and more abundant OTUs, respectively. The variation in abundance indicates that environmental changes can either reinforce or relax the negative feedback loop controlling the population growth of the core microbiome of *L. chondrodes*. Understanding how the interaction between the intrinsic and extrinsic population factors affects the abundance of different members of the sponge microbiome is essential to determine the effect of environmental change on the fitness of specific members of the microbiome. Establishing this link will help to provide mechanistic insights into the effects of climate change on sponge holobionts and predict their response to future oceanic conditions.

## Electronic supplementary material

ESM 1 (DOCX 12 kb)

ESM 2 (DOCX 3967 kb)
