# Supplementary material for: Short-Term Exposure to High-Temperature Water Causes a Shift in the Microbiome of the Common Aquarium Sponge Lendenfeldia chondrodes
Source: Microb Ecol. 2020 Aug 7;81(1):213–22. doi: 10.1007/s00248-020-01556-z (PMC7794106; doi:10.1007/s00248-020-01556-z)
Supplement: Supplementary file 2 — (DOCX 3967 kb) [file 248_2020_1556_MOESM2_ESM.docx]

**
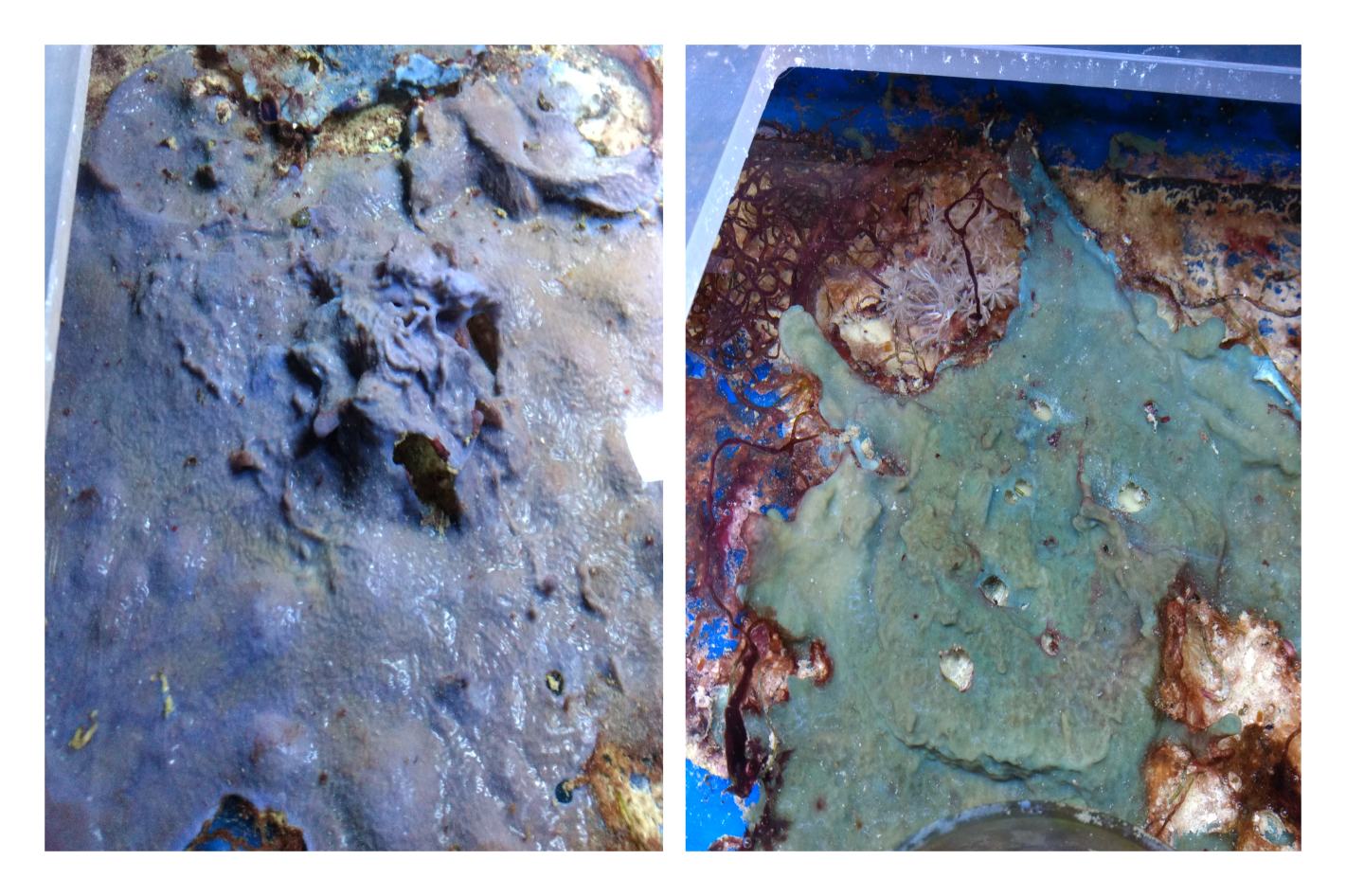
Supplementary Figure 1:** Purple (left) and green morphs of *Lendenfeldia chondrodes*. Picture not at scale. As a reference, the specimen on the left is ca. 20 cm and the specimen on the right is ca. 10 cm across.

**
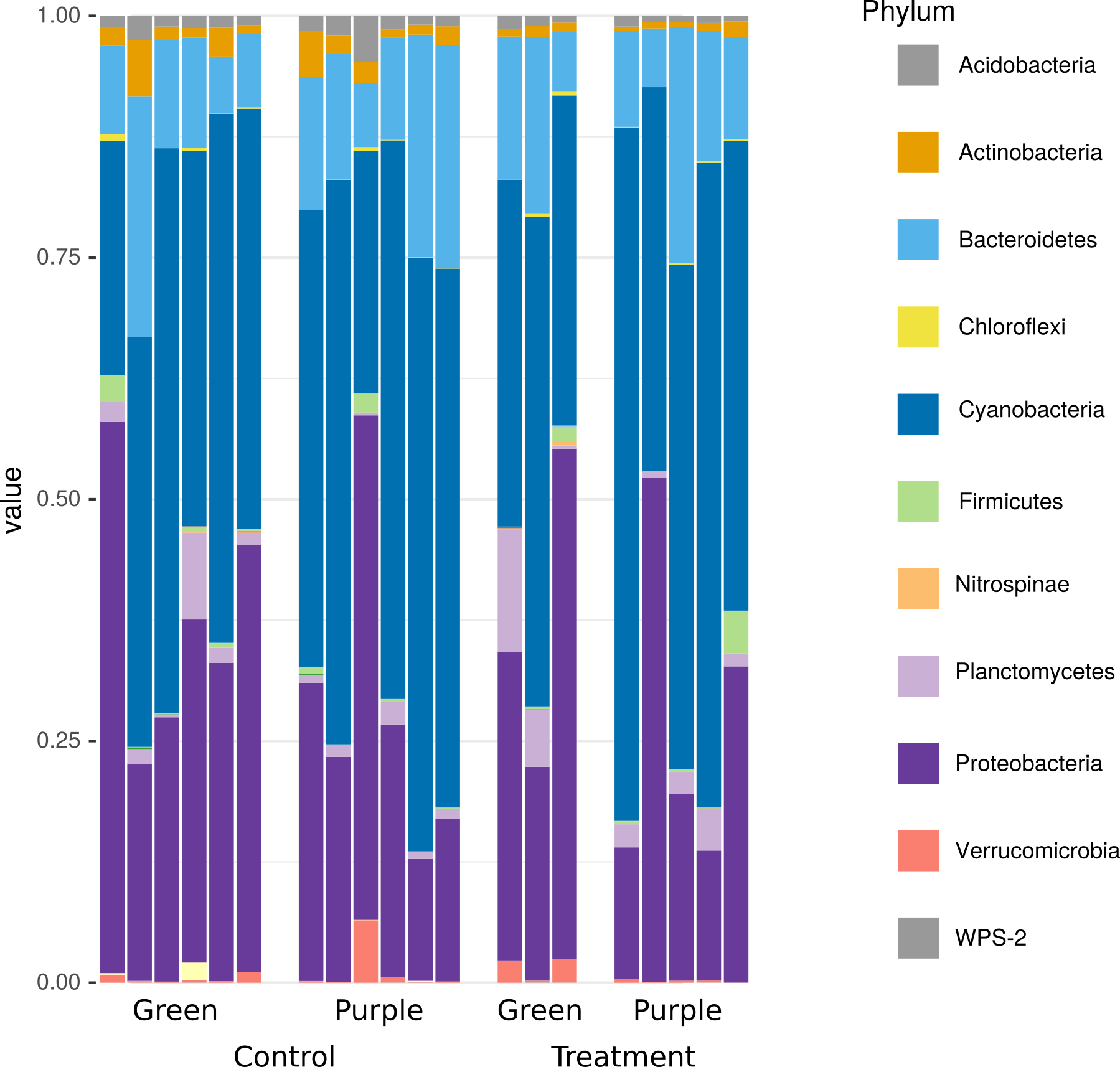
**

**Supplementary Figure 2:** Per sample relative abundance of bacterial phyla associated with the common aquarium sponge *Lendenfeldia chondrodes*. Only phyla visible in the graph are listed in the legend.


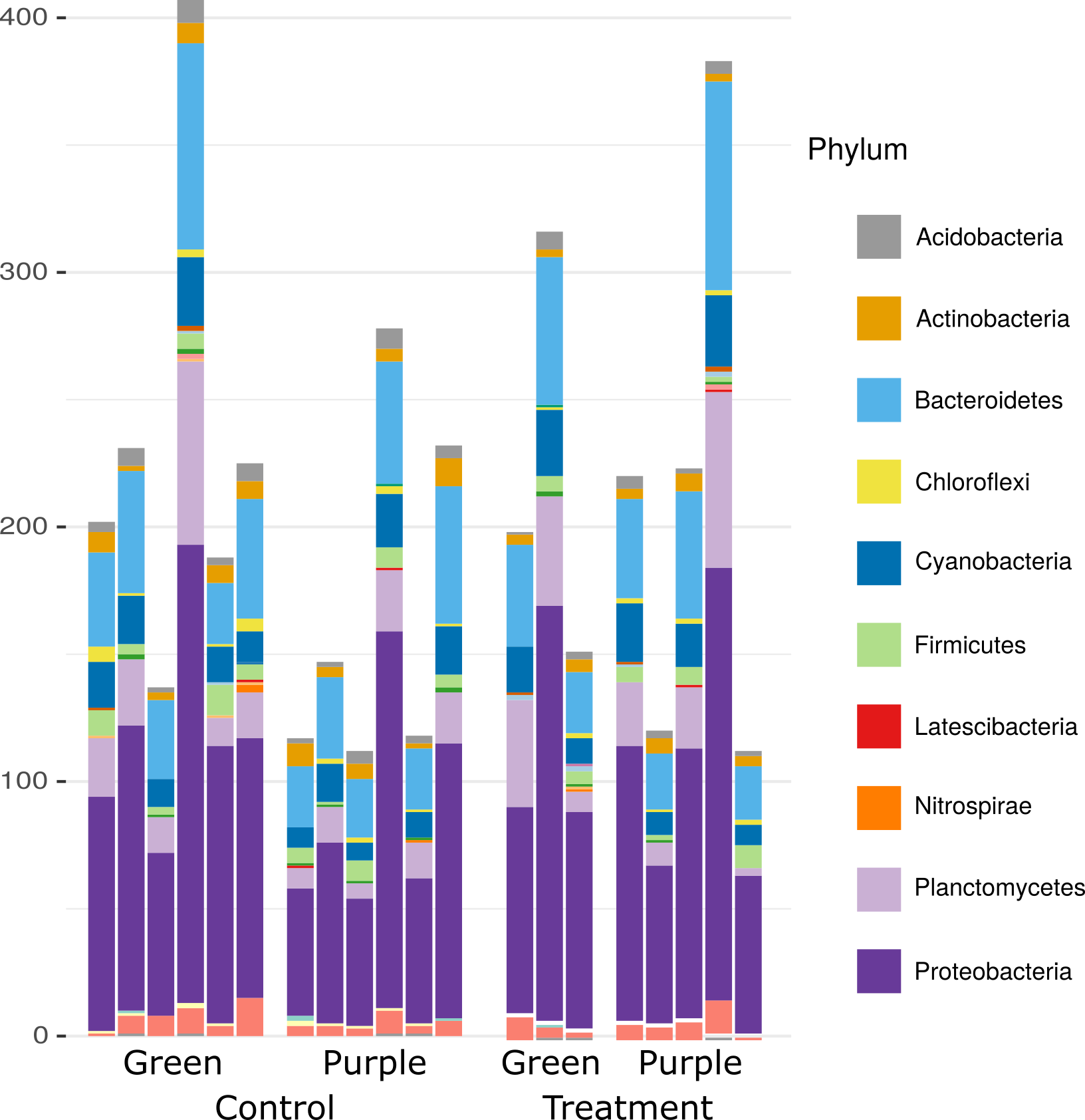


**Supplementary Figure 3:**  Per phylum bacterial richness (number of OTUs) for control and treated green and purple morphs of the common aquarium sponge *Lendenfeldia chondrodes*. Only phyla visible in the graph are listed in the legend.


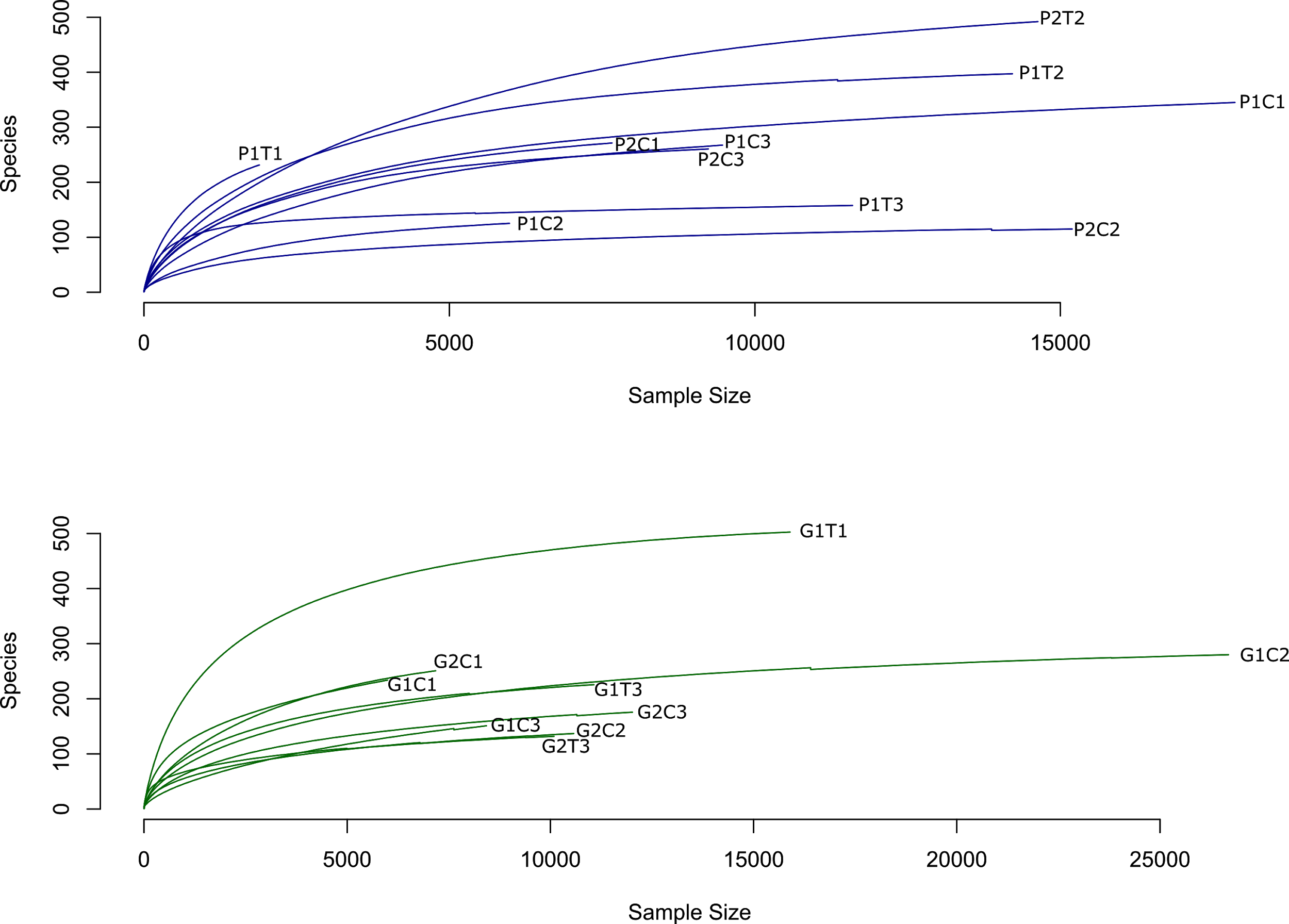


**Supplementary Figure 4:** Per sample rarefaction curves for control and treated samples of the green and purple morphs of the common aquarium sponge *Lendenfeldia chondrodes*. G=Green, P=Purple, C=Control, T=Treatment. The numbers associated with the color code (G or P) and the treatment code (C or T) refer to individual replicates and tanks, respectively.

**
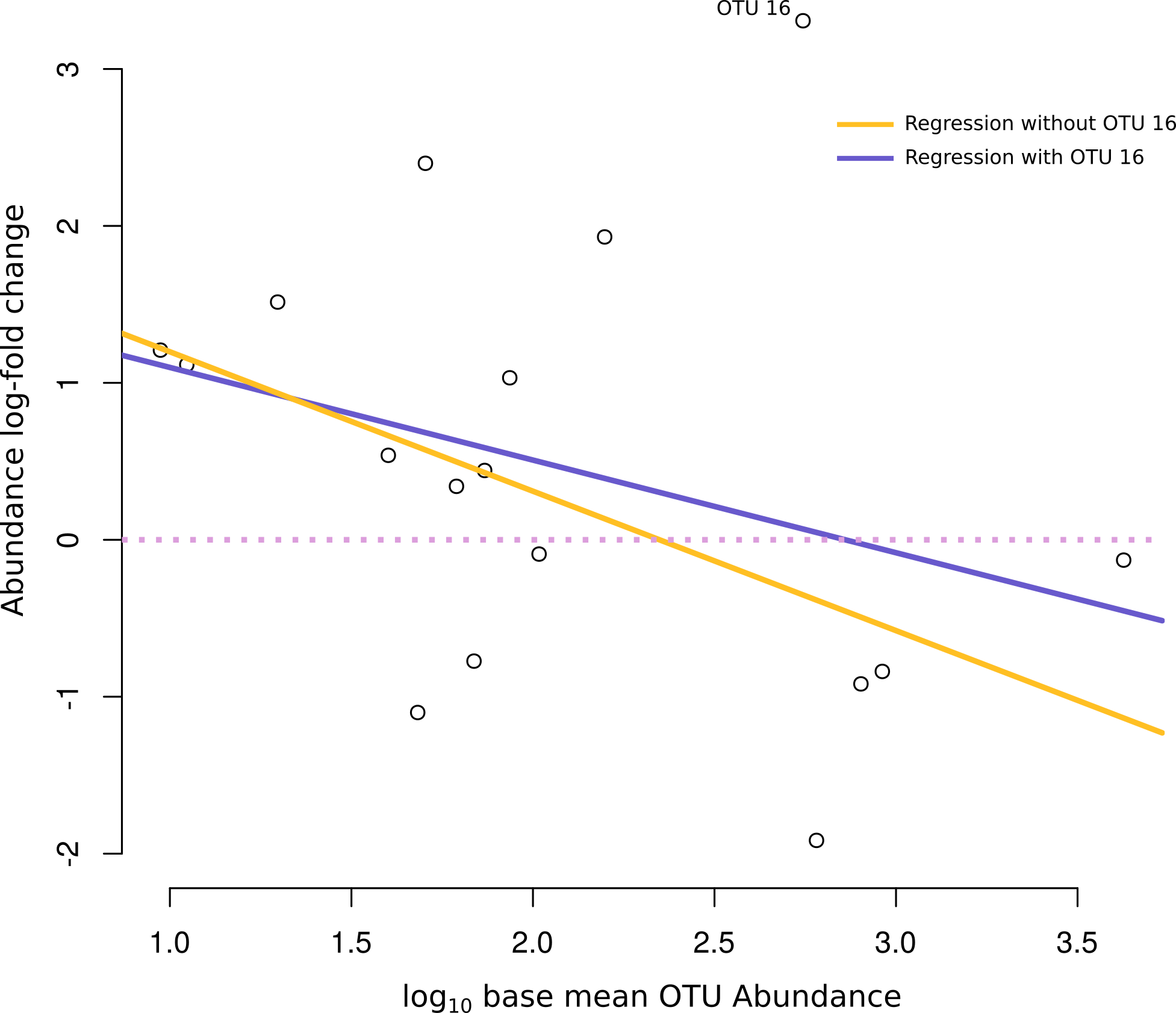
**

**
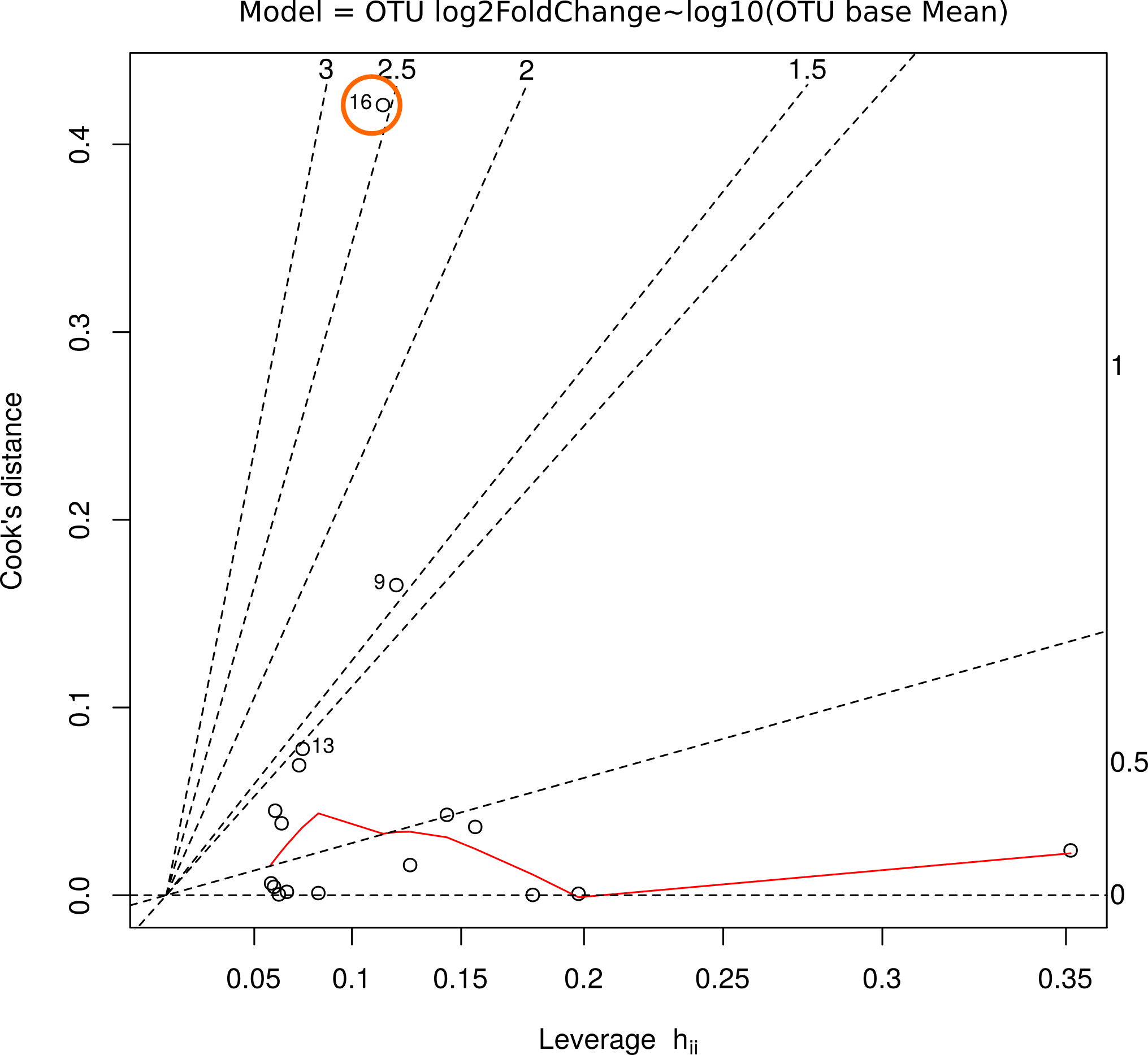
**

**Supplementary Figure 5:** (Upper panel) Regression analysis of OTU abundance log2-fold change using as predictor the base mean OTU abundance (log10 transformed). The regression line including OTU 16 is shown in purple and OTU 16 is labeled (on the top). The regression excluding OTU 16 is shown in yellow. (Lower panel) Cook’s D vs. leverage analysis showing OTU 16 as a point with high influence on the regression’s slope.
